# First Clarification of the Involvement of Glycosyltransferase MdUGT73CG22 in the Detoxification Metabolism of Nicosulfuron in Apple

**DOI:** 10.3390/plants13091171

**Published:** 2024-04-23

**Authors:** Yuefeng Zhang, Aijuan Zhao, Lijun Mu, Xiao Teng, Yingxin Ma, Ru Li, Kang Lei, Lusha Ji, Xuekun Wang, Pan Li

**Affiliations:** 1 State Key Laboratory for Macromolecule Drugs and Large-Scale Manufacturing, School of Pharmaceutical Sciences, Liaocheng University, Liaocheng 252059, China; 13562058239@163.com (Y.Z.); zaj15105403245@163.com (A.Z.); mlj15215354741@163.com (L.M.); m13021553065@163.com (Y.M.); liru0024@163.com (R.L.); leikang@lcu.edu.cn (K.L.); jilusha@lcu.edu.cn (L.J.); 2 Rizhao Research Institute of Agricultural Science, Rizhao 276500, China; teng-xiao-rzaas@hotmail.com

**Keywords:** glycosyltransferase, MdUGT73CG22, acetolactate synthase inhibitor, nicosulfuron, herbicide, detoxification mechanism

## Abstract

Nicosulfuron, an acetolactate synthase (ALS) inhibitor herbicide, is a broad-spectrum and highly effective post-emergence herbicide. Glycosyltransferases (GTs) are widely found in organisms and transfer sugar molecules from donors to acceptors to form glycosides or sugar esters, thereby altering the physicochemical properties of the acceptor molecule, such as participating in detoxification. In this study, nine glycosyltransferases in group D of the apple glycosyltransferase family I were predicted to possibly be involved in the detoxification metabolism of ALS-inhibiting herbicides based on gene chip data published online. In order to confirm this, we analysed whether the expression of the nine glycosyltransferase genes in group D was induced by the previously reported ALS-inhibiting herbicides by real-time PCR (polymerase chain reaction). It was found that the ALS-inhibiting herbicide nicosulfuron significantly increased the expression of the *MdUGT73CG22* gene in group D. Further investigation of the mechanism of action revealed that the apple glycosyltransferase MdUGT73CG22 glycosylated and modified nicosulfuron both in vivo and ex vivo to form nicosulfuron glycosides, which were involved in detoxification metabolism. In conclusion, a new glycosyltransferase, MdUGT73CG22, was identified for the first time in this study, which can glycosylate modifications of the ALS-inhibiting herbicide nicosulfuron and may be involved in the detoxification process in plants, which can help to further improve the knowledge of the non-targeted mechanism of herbicides.

## 1. Introduction

There are many types of herbicides, and they are classified in various ways. Based on the mode of action, they can be classified into selective and inactivating herbicides, with the former having different levels of resistance to different species of plants, such as gaynon, bendazone, and dinocarb [1], and the latter being toxic to all plants, as long as the green parts are in contact [2]. Herbicides can be classified according to their transmission type; the contact herbicides only kill the part of plants that come into contact with herbicides, whereas the systemic herbicides can be transported to other parts of the plant after uptake, thus poisoning the whole plant [3].

There are currently 54 ALS inhibitors used in agriculture, which belong to five subclasses: sulfonylureas, imidazolinones, triazolopyrimidines, pyrimidinylthiobenzoates, and sulfonylamino carbonyltriazolinones [4]. Among them, triazolopyrimidine sulfonamides are a new class of acetolactate synthetase (ALS) inhibitors that have been developed and rapidly applied since the 1990s. By modifying sulfonylureas, they have retained the advantage of ultra-high efficiency while solving the obvious disadvantage that some varieties have a long residual time in the soil, which can cause damage to the subsequent crop, and have thus become the first choice among the five subclasses of herbicides [5].

Nicosulfuron is a systemic herbicide that can be absorbed by weed stems, leaves, and roots and, subsequently, be conducted through the plant, causing growth stagnation, greening of stems and leaves, and gradual dieback of sensitive plants, typically within 20–25 days [6]. Plants are sensitive to sulfonylurea herbicides such as nicosulfuron. Nicosulfuron is a sulfonylurea-type endopeptic herbicide that can be quickly absorbed by the stems, leaves, and roots of plants. It inhibits the activity of acetyllactate synthase in the plant to prevent branched-chain amino acid synthesis, thereby preventing cell division and stopping the growth of sensitive plants [7]. Even if only small amounts of such herbicide residues are present in the soil, they can affect plant growth and cause a lot of harm to beneficial non-target organisms in agricultural production as well as to the environment [8]. Therefore, it is of great significance to study the reproduction and genotoxicity of herbicides for selecting safe species and protecting ecosystems.

Glycosyltransferases (GTs) are a class of highly divergent multisource gene families present in almost all organisms that catalyse biological transformation reactions, transferring sugar groups from activated donor molecules to acceptor molecules [9]. In plants, UDP-glucose is the more abundant sugar donor, in addition to UDP-galactose, UDP-rhamnose, UDP-xylose, and UDP-glucuronic acid [10]; whereas glycosyl acceptors include non-carbohydrates, such as proteins, lipids, antibiotics, steroids, phenolics, terpenes, cyanohydrols, plant hormones, and other substances, in addition to monosaccharides, oligosaccharides, polysaccharides, cyanoalcohols, phytohormones, alkaloids, phytotoxins, and exogenous substances (e.g., herbicides and insecticides), etc. The site of glycosylation is mostly at the O-atom of the receptor molecule and, to a lesser extent, at the N-, S-, and C-atoms, generating the corresponding glycosides or glycolides [11], which alter the physicochemical properties of the receptor molecule, such as their hydrophilicity, chemical stability, biologically activity, and subcellular localisation, contributing to their intracellular and intraorganismal transport and storage, etc. [12].

Taylor et al. showed that when wheat (*Triticum aestivum* L.) plants were spray-treated with three safener chemistries, namely cloquintocet mexyl, mefenpyr diethyl, and fenchlorazole ethyl, a large number of glycosyltransferase genes were induced to be expressed [13]. Furthermore, it has been claimed that several *Arabidopsis* (*Arabidopsis thaliana* (L.) *Heynh*) UGTs are involved in the metabolic detoxification of the exogenous substances 2,4,5-trichlorophenol and 3,4-dichloroaniline (DCA) [14,15]. In addition, *Arabidopsis* (*Arabidopsis thaliana* (L.) *Heynh*) plants have been shown to have enhanced detoxification of 2,4-dihydroxy-1,4-benzoxazin-3-one (DIBOA) and 2,4-dihydroxy-7-methoxy-1,4-benzoxazin-3-one (DIMBOA) by overexpression of either the *GT BX8* or *GT BX9* genes [16]. Su et al. identified and cloned an enzyme with O-glycosyltransferase activity, UGT73A17, which was responsible for the biosynthesis of several flavonoid glycosides, and it was also involved in the thermal response and quality of tea (*Camellia sinensis* (L.) *O. Ktze*) [17]. Li et al. identified 229 members of family 1 through a genome-wide analysis of the apple UGTs, which were clustered into 18 groups, from A to R [18]. However, there are fewer studies on the direct involvement of glycosyltransferases in the detoxification metabolism of plant pesticides, and the involvement in the detoxification metabolism mechanism of the ALS-inhibiting herbicide nicosulfuron has not been reported. Therefore, it is important to study the involvement of glycosyltransferases in the mechanism of pesticide detoxification metabolism in plants. In this study, we identified a new apple (*Malus* × *domestica Borkh.*) glycosyltransferase MdUGT73CG22 by molecular biology techniques, which can glycosylatively modify the ALS-inhibiting herbicide nicosulfuron and may be involved in the detoxification process in plants, which can help to further improve the knowledge on the mechanism of non-targeted action of herbicides.

## 2. Results

### 2.1. Candidate Gene MdUGT73CG22 Identified

**Figure 1:**
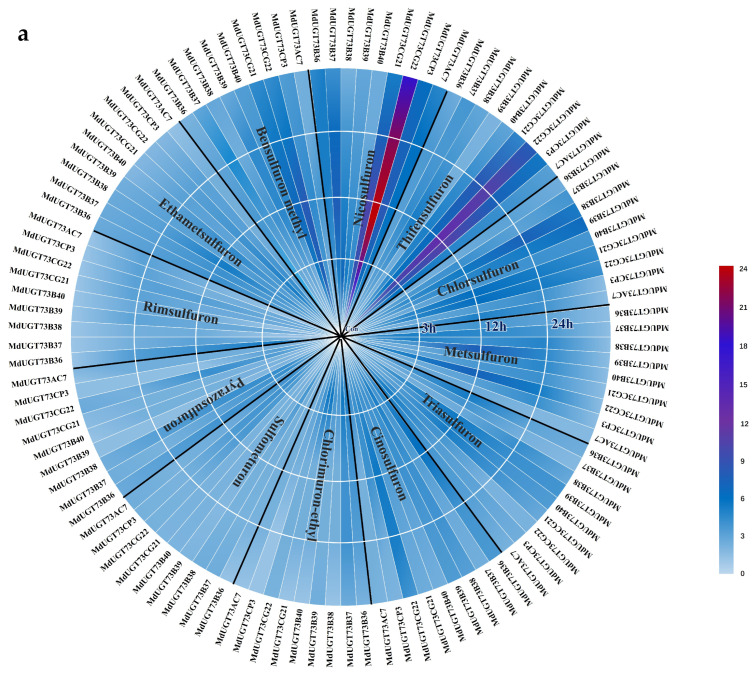

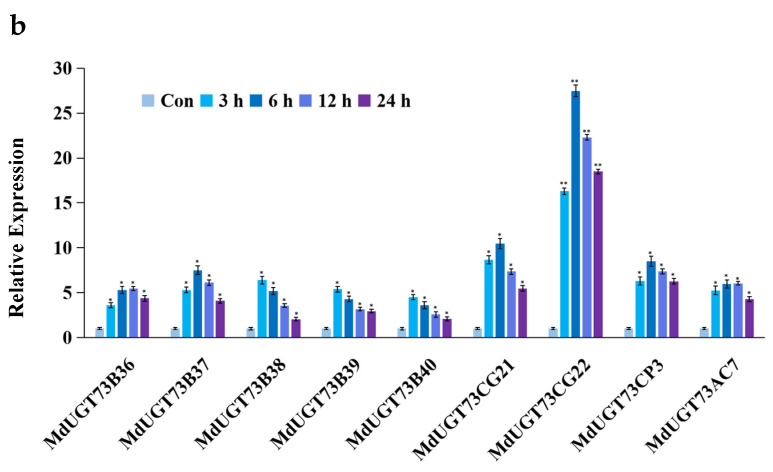
Relative expression of nine glycosyltransferase genes induced by 12 types of acetolactate synthase (ALS) inhibitor herbicides: (**a**) real-time PCR detection of glycosyltransferase gene *MdUGT73B40* (*MD05G1085700*), *MdUGT73CG21* (*MD12G1104300*), *MdUGT73CG22* (*MD12G1104800*), *MdUGT73CP3* (*MD05G1085400*), and *MdUGT73AC7* (*MD05G1085600*) are affected by acetolactate synthase (ALS) inhibitor herbicides: bensulfuron methyl, nicosulfuron, thifensulfuron, chlorsulfuron, metsulfuron, triasulfuron, cinosulfuron, chlorimuron-ethyl, sulfometuron, pyrazosulfuron, rimsulfuron, and ethametsulfuron induced expression at 0, 3, 6, 12, and 24 h; (**b**) real-time PCR detection of glycosyltransferase gene *MdUGT73CG22* (*MD12G1104800*) subjected to expression induced by the acetolactate synthase (ALS) inhibitor-like herbicide nicosulfuron at 0, 3, 6, 12, and 24 h. Note: * p < 0.05, ** p < 0.01, *n* = 3.

Based on the gene chip database published on NCBI (https://www.ncbi.nlm.nih.gov/, accessed on 18 June 2023) and the apple (*Malus* × *domestica Borkh.*) glycosyltransferase phylogenetic tree [18], nine glycosyltransferase genes *MdUGT73B36* (*MD17G1100000*), *MdUGT73B37* (*MD17G1100300*), *MdUGT73B38* (*MD05G1086300*), *MdUGT73B39* (*MD05G1085800*), *MdUGT73B40* (*MD05G1085700*), *MdUGT73CG21* (*MD12G1104300*), *MdUGT73CG22* (*MD12G1104800*), *MdUGT73CP3* (*MD05G1085400*), and *MdUGT73AC7* (*MD05G1085600*) were predicted to possibly be involved in the detoxification metabolism of ALS-inhibiting herbicides. To validate their functional predictions, we treated wild-type apple (*Malus* × *domestica Borkh.*) seedlings with herbicides, including bensulfuron methyl, nicosulfuron, thifensulfuron, chlorsulfuron, metsulfuron, triasulfuron, cinosulfuron, chlorimuron-ethyl, sulfometuron, pyrazosulfuron, rimsulfuron, and ethametsulfuron, and real-time PCR was performed to detect the mRNA expression levels of these nine glycosyltransferase genes, respectively. The results are shown in Figure 1 and Table S1; compared with other ALS-inhibiting herbicides and glycosyltransferase genes, nicosulfuron significantly increased the mRNA expression level of glycosyltransferase gene *MdUGT73CG22* (*p* < 0.01). For this reason, we chose the apple (*Malus* × *domestica Borkh.*) glycosyltransferase gene *MdUGT73CG22* as a candidate gene in this study.

### 2.2. Analysis of Apple Glycosyltransferase MdUGT73CG22 Tissue Expression Pattern

**Figure 2:**
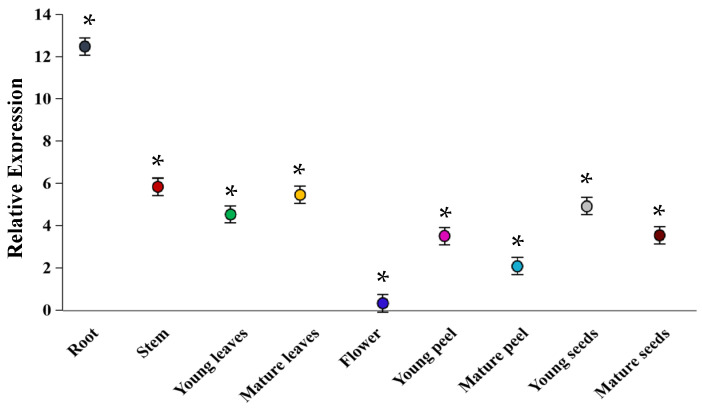
Real-time PCR detection of apple glycosyltransferase MdUGT73CG22 tissue expression pattern, including root, stem, young leaves, mature leaves, flower, young peel, mature peel, young seeds, and mature seeds. Note: * *p* < 0.05, *n* = 3.

In order to explore the expression of gene *MdUGT73CG22* in different developmental periods of apple (*Malus* × *domestica Borkh.*), RNA was extracted from fresh materials of different growth and developmental periods of apple (*Malus* × *domestica Borkh.*) and reversed transcribed into cDNA; then, the mRNA expression level of *MdUGT73CG22* was detected by real-time PCR. The results are shown in Figure 2; the gene *MdUGT73CG22* was expressed at the highest level in apple (*Malus* × *domestica Borkh.*) roots (*p* < 0.05) and the lowest level in flowers (*p* < 0.05). This result suggests that the apple glycosyltransferase gene *MdUGT73CG22* may play a primary role in the root system.

### 2.3. Identification of Substrates for Glycosylation by Apple Glycosyltransferase MdUGT73CG22

**Figure 3:**
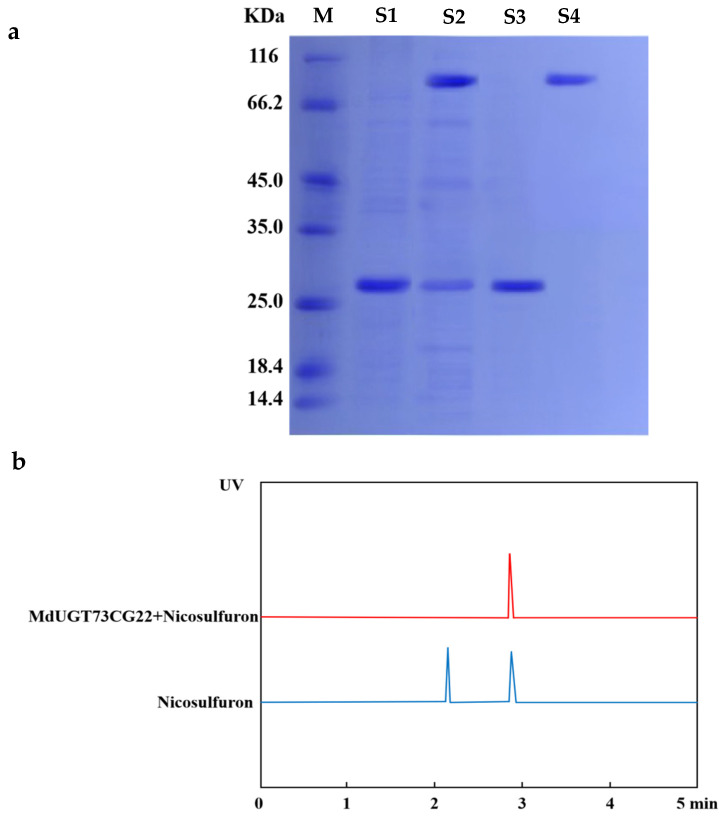

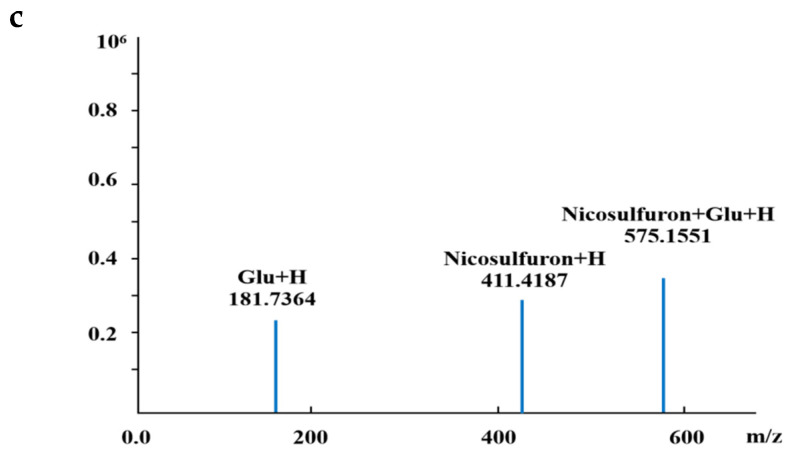
HPLC identification of glycosylation substrates for the apple glycosyltransferase MdUGT73CG22: (**a**) lane M is Mark; lane S1 is GST tag protein extracted from bacterial solution; lane S2 is MdUGT73CG22-GST fusion protein extracted from bacterial solution; lane S3 is purified GST tag protein; lane S4 is purified MdUGT73CG22 protein. Induced expression of apple glycosyltransferase MdUGT73CG22 protein GST tag: 26.0 KDa; MdUGT73CG22-GST: 81.0 KDa; (**b**) HPLC assay revealed that apple glycosyltransferase MdUGT73CG22 could modify nicosulfuron by glycosylation in vitro, with a peak time of 2.9 min for nicosulfuron and 2.3 min for nicosulfuron glycosides; (**c**) determination of nicosulfuron glycosides, the product of apple glycosyltransferase MdUGT73CG22, by molecular weight by HPLC-MS.

Glycosyltransferase is a kind of modified enzyme; in order to further test its activity, we induced and purified the MdUGT73CG22 enzyme protein in vitro and analyzed the total protein and purified protein after induction by SDS-PAGE, which showed that the amount of the total protein after induction was significantly increased at 81.0 kDa compared with the amount before induction, and the purified protein band appeared was close to 81.0 kDa (the target band carrying the GST tag, which was 26.0 kDa) (Figure 3a), indicating that the MdUGT73CG22 protein was successfully expressed and purified.

In order to detect the activity of MdUGT73CG22, we used HPLC (high performance liquid chromatography) to detect the reaction products of MdUGT73CG22 protein. The results are shown in Figure 3b,c. MdUGT73CG22 catalysed nicosulfuron to nicosulfuron glycoside within 30 min; however, in the control experiments, no nicosulfuron glycoside production was found. This shows that MdUGT73CG22 can use glucose as a glycosyl donor to glycosylate nicosulfuron to produce nicosulfuron glycoside in vitro.

### 2.4. Acquisition of the MdUGT73CG22 Transgenic Apple Line and Identification of the Detoxification Function

**Figure 4:**
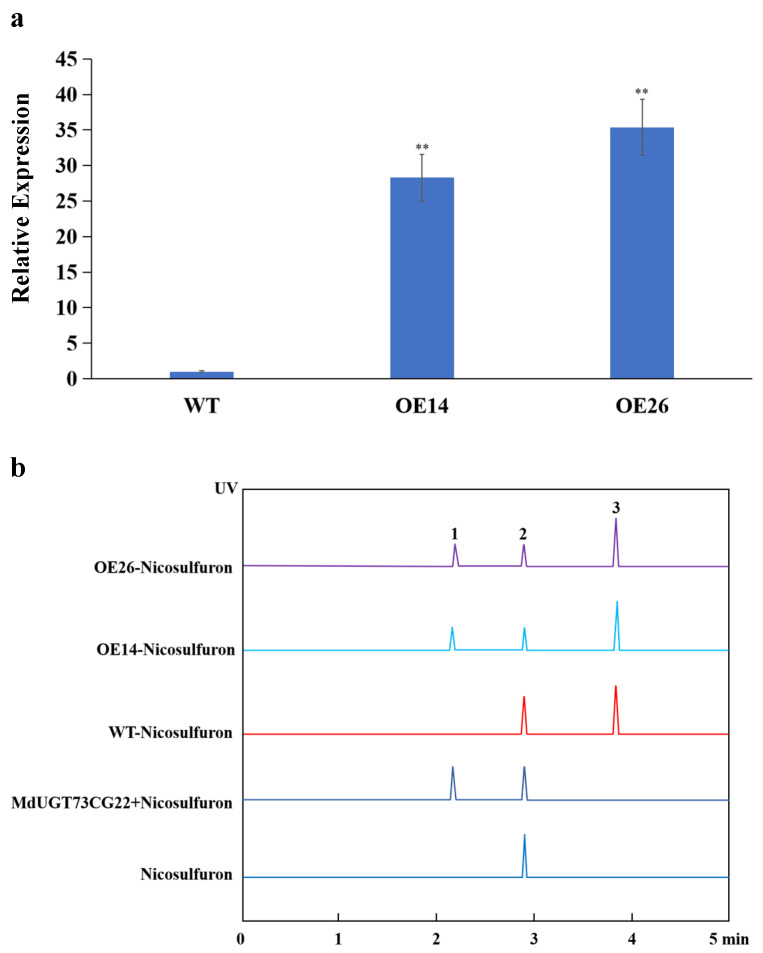
HPLC detection of nicosulfuron glycosides in apple glycosyltransferase gene *MdUGT73CG22* overexpression lines *OE14* and *OE26*: (**a**) expression level of gene *MdUGT73CG22* in overexpression lines detected by real-time PCR; (**b**) HPLC detection of nicosulfuron glycosides in *MdUGT73CG22* overexpression lines *OE14* and *OE26*. Note: 1, nicosulfuron glycosides, 2.3 min; 2, nicosulfuron, 2.9 min; 3, internal reference, 3.8 min. Note: ** *p* < 0.01, *n* = 3.

In the above study, we found that the MdUGT73CG22 enzyme protein could catalyse nicosulfuron in vitro. Could MdUGT73CG22 also catalyse nicosulfuron in apple (*Malus* × *domestica Borkh.*)? We extracted the (*Malus* × *domestica Borkh.*) apple RNA, reverse transcribed it into cDNA, successfully cloned the *MdUGT73CG22* gene by PCR amplification, constructed the plant overexpression vector of *MdUGT73CG22*, and successfully obtained the *MdUGT73CG22* overexpression strains *OE14* and *OE26* through the mediation of *R. rhizogenes* strain K599 (Figure 4a). Then, we treated each line with an appropriate amount of nicosulfuron and extracted the total glycosides; the results showed that the content of nicosulfuron glycosides in the *MdUGT73CG22* overexpression lines were significantly increased compared with the control (Figure 4b). These results suggest that the MdUGT73CG22 enzyme protein can also catalyse nicosulfuron in vivo and convert it to nicosulfuron glycosides. Taken together, the results show that the apple (*Malus* × *domestica Borkh.*) glycosyltransferase MdUGT73CG22 can modify the ALS-inhibiting herbicide nicosulfuron by glycosylation, which may be involved in the detoxification process of plants and help to further improve the knowledge of the mechanism of non-target action of herbicides.

## 3. Discussion

Pesticides are effective in safeguarding agricultural production, controlling pests, diseases, and weeds, and achieving the goal of increasing crop production and generating income. However, excessive use of pesticides causes serious pollution and damage to the environment and ecology, which in turn threatens the entire food chain and human health [19]. Therefore, it is of great theoretical significance and practical value to study the metabolism and detoxification mechanism of pesticides in crops, reduce the accumulation of pesticides in crops, and ensure the safety and quality of agricultural products.

The degradation of pesticides usually involves hydrolysis, photolysis, and enzymatic degradation [20,21]. Pesticides can be biodegraded in plants through various metabolic and transformation pathways [22]. Studying its mechanism of action will help to clarify the composition of pesticide residues in agricultural commodities and to assess the dietary risk of pesticides. Meanwhile, it also can provide an important basis for the creation of new pesticides and the safe use of pesticides. The results of the related research can be an important scientific basis in the fields of crop breeding, transgenic plant engineering, and phytoremediation of environmental pollution.

The metabolic transformation of pesticides in plants is mainly divided into three stages. Phase I metabolism is the enzyme-catalysed reaction and abiotic degradation process, including oxidation, hydrolysis, and reduction [23]. In general, this process converts pesticides into more water-soluble compounds with lower phytotoxicity, while in some special cases, more phytotoxic substances may be generated, such as the metabolic activation of some herbicides in plants [24]. Phase II metabolism involves the conjugation of pesticides and their metabolites with plant endogenous substances including carbohydrates, amino acids, and glutathione [25]. The plant enzymes involved in phase II metabolism include glutathione S-transferases (GSTs), GTs, and malonyltransferases (MTs), among others [26]. Enzymes have substrate specificity; therefore, GTs can selectively search for their own substrate and combine with it. The conjugates formed in phase II metabolism are usually more water-soluble than the parent compound, have lower phytotoxicity, and can be stored in organelles, such as vesicles [27]. Phase III metabolism actively removes conjugates and associated catabolic metabolites formed by the phase II metabolism from the cytosol, mainly through adenosine triphosphate (ATP)-dependent transporter proteins, and deposits them into vesicles for temporary storage or further catabolic metabolism [28]; some conjugates are transported to other regions of plant cells, such as aggregation into the cell walls [27]. There are also some reports that classify some of the processes of phase III metabolism as phase IV metabolism, including the re-transport of vesicular products of phase III metabolism back into the cytosol for mineralisation and the incorporation of metabolic intermediates into the conjugated residues, among others [29,30,31,32]. However, at present, the definition of phase IV metabolism is not clear, and opinions are not consistent. In addition, the same pesticide can undergo different pathways of metabolism simultaneously in the same plant.

Numerous studies have shown that GTs play an important role in pesticide detoxification and plant selectivity to herbicides [33,34,35,36]. Zhang et al. investigated the metabolism of atrazine after overexpression of the *ARGT1* gene in rice (*Oryza × sativa*) and found that the glycosyltransferase 1 (ARGT1) played an important role in the metabolism and detoxification of atrazine in transgenic rice (*Oryza × sativa*). GTs were involved in the detoxification process of atrazine in *alfalfa Medicago sativa*, which was treated with atrazine, showed increased activities of GTs and GSTs (Glutathione S-transferase), and detected two O-glycosylated metabolites and homoglutathione (hGSH) conjugates [33]. Huang et al. found that *Arabidopsis thaliana* AtUGT91C1 played an important role in herbicide detoxification by glycosylating sulforaphane [34]. Yu et al. found that glutathione could reduce chlorothalonil (CHT) residues in tomato (*Lycopersicon esculentum*) by inducing the expression of the UDP-glycosyltransferase (UGT) gene [35]. Brazier- Hicks et al. reported that 44 GTs from the model plant *Arabidopsis thaliana* catalysed the O-glycosylation of the pesticide synthesis intermediate chlorophenols, with UGT72B1 also showing N-glycosylation activity towards chloranilides [36].

Individual genotypes of the cultivated apple tree (*Malus domestica Borkh.*) differ considerably in their fruit and vegetative characteristics. The results of apple genomic studies confirmed that cultivated apple varieties originate mainly from *Malus sieversii M. Roem* found in Central Asia. Other species such as the European apple (*Malus sylvestris Mill.*), the small-fruited *Malus baccata* (L.) *Borkh.* from Northeast Asia, and the Eastern Caucasian apple *Malus orientalis Uglitz.* also contributed to the creation of today’s cultivated varieties. This heterogeneity can manifest itself when working with seedlings created by generative propagation, which is part of the methodical procedure. Therefore, the role of the glycosyltransferase MdUGT73CG22 identified in this study in the detoxification metabolism of nicosulfuron in apples (*Malus* × *domestica Borkh.*) may have species preference. In the following research, we will try to expand the sample size and elucidate the detoxification function of glycosyltransferase MdUGT73CG22 in different apple varieties.

In this paper, the metabolic mechanism of the apple (*Malus* × *domestica Borkh.*) glycosyltransferase gene regulating the detoxification of the acetolactate synthase (ALS) inhibitor herbicide nicosulfuron was investigated, using nicosulfuron as a test pesticide and molecular biology techniques, which resulted in the first discovery of a novel apple (*Malus* × *domestica Borkh.*) glycosyltransferase, MdUGT73CG22, which can glycosylate ALS-inhibiting herbicide nicosulfuron and may be involved in the plant detoxification process. This helps us to better understand and utilize herbicides, which are convenient and do not cause pollution to the environment or damage the plants themselves. This study will lay the foundation for subsequent biological detoxification research.

## 4. Materials and Methods

### 4.1. Plant Material

We selected the roots, stems, leaves, flowers, and fruits of the 10-year-old *Malus × domestic Borkh.* apple tree planted by the School of Life Sciences, Shandong Agricultural University. Twenty samples were randomly collected from each plant, with a total of 10 strains collected. Please refer to “Apple Germplasm” in the “Resource Description Specification and Data Standards” [37] for details. The samples were frozen in liquid nitrogen and stored at −80 °C for expression pattern analysis of the glycosyltransferase gene *MdUGT73CG22*.

Transgenic apple (*Malus* × *domestica Borkh.*) lines (*MdUGT73CG22-OE14* and *MdUGT73CG22-OE26*) were obtained by transforming the leaves of Gala using tissue culture seedlings of the clone apple (*Malus* × *domestica Borkh.*) “GL-3”. Tissue culture seedlings were grown at 22 °C, 60 µmol·m^−2^·s^−1^, and 14/10 h photoperiod. After 30 d of succession culture and 45 d of rooting medium culture, the seedlings were incubated for 1 week, and then the tissue-cultured seedlings were transferred to plastic pots (8.5 × 8.5 × 7.5 cm^3^) and incubated in a light incubator.

### 4.2. Treatment of Apple Seedlings with ALS-Inhibiting Herbicides

The stratified apple (*Malus* × *domestica Borkh.*) seeds were placed in a light incubator at 25 °C with 14 h of light and 10 h of darkness until the root length reached 3 cm; they were then transplanted into a 1:1 soil of nutrient soil and vermiculite and cultured until the six-leaf stage. A certain amount of acetolactate synthase (ALS) inhibitor herbicides (Table 1) was weighed separately, dissolved in DMSO, and prepared to a final concentration of 10 μmol/L by dilution with ddH_2_O water. Then, 50 mL of each compound solution was taken and placed in a petri dish, the roots of the 6-leaf stage apple (*Malus* × *domestica Borkh.*) seedlings were placed in the solution, the plant materials were taken at five time points, 0 h, 3 h, 6 h, 12 h and 24 h, respectively, and they were wrapped in tin foil and labelled to be placed in a refrigerator at −80 °C for subsequent RNA extraction.

### 4.3. Real-Time PCR

For RNA extraction, we referred to the instructions of the plant RNA kit polysaccharide polyphenol/complex plant RNA rapid extraction kit, as SPARKscript II All-in-one RT SuperMix (AC0307, Sparkjade, Jinan, China).

For cDNA reverse transcription, we referred to the instructions of the reverse transcription kit, as 2 × Spark Taq PCR Master Mix (AG0305, Sparkjade, Jinan, China).

RT-qPCR was performed using SYBR Green PCR Master Mix kit (Q111-02, VazymE, Nanjing, China) by Bio-Rad Thermal Cycling System (CFX Connect, Hercules, CA, USA). The expression level of the reference gene β-actin was standardised using the ΔΔCt method to normalise the expression levels; the primer sequences are shown in Table S2, and the nucleotide and amino acid sequences of MdUGT73CG22 are shown in Table S3.

### 4.4. Expression Vector Construction

Pfu DNA Ploymerase (PC1300, Solepol Biotechnology Co., Shanghai, China) was used to amplify the target fragment from the apple (*Malus* × *domestica Borkh.*) seedling cDNA; then, the target fragment was recovered using an agarose gel DNA recovery kit (ZY511-100, Runye Biotechnology Co., Shanghai, China). The gel-recovered product was ligated into a pMD18-T vector, transformed into *E. coli* DH5α, and screened for recombinant plasmids by the blue–white spot method. The recombinant plasmid was double digested to identify the positive clones and sent to BGI Genomics Co., Ltd. (Shenzhen, China) for sequencing of the target fragments. Here, 100% of the sequenced fragments were correctly sequenced; then, the target gene *MdUGT73CG22* was recombinantly transferred into the prokaryotic expression vector pGEX-2T and the plant expression vector PBI121, respectively. After bacteriophage PCR and double enzyme digestion verification, the positive plasmids *MdUGT73CG22*-pGEX-2T (Figure S1a) and *MdUGT73CG22*-PBI121 (Figure S1b) were transfected into the protein expression strains *E. coli* BL-21 and *R. rhizogenes* strain K599, respectively, for the subsequent protein purification and the obtainment of transgenic strains, respectively.

### 4.5. Protein Purification and In Vitro Enzymatic Reactions

Protein purification: single bacteria containing *MdUGT73CG22*-pGEX-2T were inoculated into LB liquid medium at 200 rpm/min and incubated at 37 °C overnight. The activated strain was inoculated in 100 mL LB medium containing kanamycin (50 μg/mL) at a ratio of 1:50, and when the OD_600_ value of the bacterial solution was about 0.6, the bacterial solution was inoculated with a final concentration of 0.4 mmol/L of Isopropyl-beta-D-thiogalactopyranoside (IPTG), at a final concentration of 0.4 mmol/L. Soluble recombinant protein expression was induced with Isopropyl-beta-D-thiogalactopyranoside (IPTG) and incubated at 20 °C, 200 rpm/min, for 24 h. The induced bacterial fluids were subjected to target protein purification according to the instructions of the Beyotime GST-tag Protein Purification Kit (P2262, Beyotime, Shanghai, China).

In vitro enzymatic reaction: the protein obtained by purification as described above was subjected to an in vitro enzymatic reaction with the target compounds in a specific system, consisting of 10 µL of 0.5 M Tris-HCl (pH 8.0), 5 µL of 50 mM MgSO_4_, 5 µL of 200 mM KCl, 2.5 µL of 0.1 M UDP-glucose, 1 µL of 10% β-mercaptoethanol, 1 µL of 100 mM nicosulfuron, 2 µL MdUGT73CG22 protein, and ddH_2_O, and the volume was made up to 100 µL by adding ddH_2_O.

### 4.6. Transgenic Apple Seedling Screening

The test material used for apple genetic transformation was the tissue culture seedlings of clone “GL-3”, with strong regenerative ability and high transformation efficiency, obtained from the “Gala” apple. The leaves of “GL-3” were transformed with *R. rhizogenes* strain K599, which carries the sequence *MdUGT73CG22*-PBI121, and 50 mg·L^−1^ kanamycin and 250 mg·L^−1^ sodium cefotaxime were added to the MS medium for the selection of regenerating shoots. During the selection process, slow-growing and yellowing regeneration shoots were discarded, and normal-growing regeneration shoots continued to be cultured on the selection medium until they developed into robust individuals with stem and leaf tissues.

DNA was extracted from “GL-3” and transgenic apple plants using the PureLink™ Genomic Plant DNA Purification Kit (K183001, Thermo Fisher Scientific, Waltham, MA, USA). The partial sequence of the CaMV 35S promoter on the vector was used as the upstream primer, the downstream primer was designed with the sequence of *MdUGT73CG22* gene for PCR amplification, and electrophoresis was carried out to detect whether it was the target gene or not.

### 4.7. Endogenous Glycoside Extraction from Transgenic Apple Seedlings

Five- to six-week-old wild-type and successful transgenic apple (*Malus* × *domestica Borkh.*) seedlings treated with the target compounds for 12 h at a final concentration of 10 µmol/L were frozen in liquid nitrogen, powdered, and homogenised in 10 mL of 90% (*v*/*v*) methanol with the internal standard. After centrifugation at 10,000× *g* for 10 min, the supernatant was transferred to a new centrifuge tube, the remaining precipitate was re-extracted with 100% (*v*/*v*) methanol and centrifuged at 10,000× *g* for 10 min, the supernatants were combined into the supernatant centrifuge tube from the previous step, and the combined supernatant was dried by heating in a fast vacuum (60 °C). The residue was resuspended in 500 μL of 20% (*v*/*v*) trichloroacetic acid and sonicated for 10 min; then, it was extracted with 2 volumes of ethyl acetate-cyclopentane-isopropanol (50:50:1). The organic phase containing fumetsulfuron was then dried by heating in a rapid vacuum (60 °C). The residue was dissolved in 100 μL of aqueous solution containing 10% MeOH (*v*/*v*) and 0.1% TFA (*v*/*v*) and vortexed for 1 min. The fraction can be used for HPLC analysis.

### 4.8. High Performance Liquid Chromatography (HPLC) Analysis of Target Products

The HPLC detection conditions were as follows: Agilent 1290-6470A ultra performance liquid chromatography–tandem mass spectrometry, Agilent (G4261B, Agilent, Santa Clara, CA, USA); Poroshelll20EC-C18 column (2.1 mm × 50 mm, 1.9 μm); column temperature: room temperature; detection wavelength: 241 nm; mobile phase A: 0.2% formic acid in water; mobile phase B: acetonitrile. The flow rate was 0.3 mL/min; the injection volume was 5 μL.

The gradient elution conditions were as follows: 0.00 min, phase A 95%, phase B 5%; 0.50 min, phase A 95%, phase B 5%; 1.60 min, phase A 20%, phase B 80%; 3.00 min, phase A 10%, phase B 90%; 4.00 min, phase A 10%, phase B 90%; 4.10 min, phase A 95%, phase B 5%; 4.50 min, phase A 95%, phase B 5%.

The mass spectrometry conditions were as follows: electrospray ion source, positive ion ionisation mode (ESI+), drying gas temperature 325 °C, drying gas flow rate 10 L/min. nebulising gas pressure 50 psi, sheath gas temperature 400 °C, sheath gas flow rate 12 L/min, capillary voltage 4000 V, nozzle voltage 500 V; data acquisition was performed in multiple reaction monitoring mode.

Mass Spectrometer: retention time 2.09 min; parent ion 411.1; daughter ion 214 and 182, cone voltage 106 VL; collision energy 14 and 20 eV.

### 4.9. Data Statistics and Analyses

All experiments were performed with at least three independent biological replicates, and each measurement was repeated three times. Data are expressed as means SD. Data were statistically analysed using Student’s *t*-test. * indicates significant differences (* *p* < 0.05, ** *p* < 0.01) relative to wild-type or controls in different experimental treatments.

## Figures and Tables

**Table 1:** Specific information on the acetolactate synthase (ALS) inhibitor class of herbicides.

Chemical Compound	Structure Name	Structural Formula	Manufacturer	CAS
Bensulfuron methyl	[3-(4,6-dimethoxypyrimidin-2-yl)-1-(2-methoxycarbonylbenzyl)sulfonylurea]	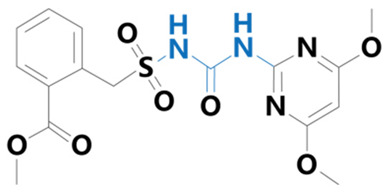	Bermuda Biotechnology Co. (Nanjing, China)	83055-99-6
Nicosulfuron	[2-(4,6-dimethoxypyrimidin-2-ylcarbamoylsulfamoyl)-N,N-dimethylnicotinamide]	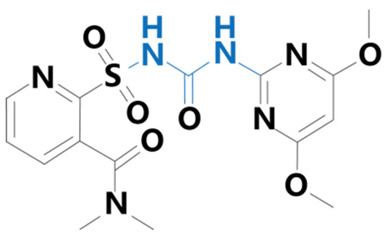	BioLabs Technology Ltd. (Beijing, China)	111991-09-4
Thifensulfuron	[3-(4-methoxy-6-methyl-1,3,5-triazin-2-ylaminocarbonylsulfamoyl)-thiophene-2-carboxylic acid]	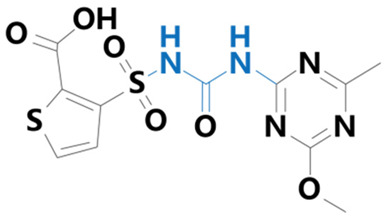	Wittig Chemical Technology Co. (Zhengzhou, China)	79277-67-1
Chlorsulfuron	[1-(2-Chlorophenylsulfonyl)3-(4-methoxy-6-methyl-1,3,5-triazin-2-yl)urea]	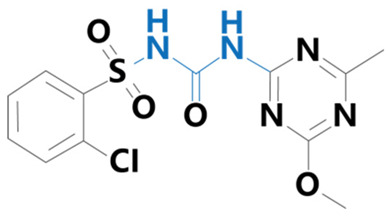	BioLabs Technology Ltd. (Beijing, China)	64902-72-3
Metsulfuron	[3-(4-methoxy-6-methyl-1,3,5-triazin-2-yl)-1-(2-formylphenyl)sulfonylurea]	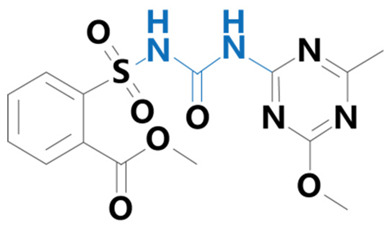	BioLabs Technology Ltd. (Beijing, China)	142469-14-5
Triasulfuron	[1-[2-(2-chloroethoxy)phenylsulfonyl]-3-(4-methoxy-6-methyl-1,3,5-triazin-2-yl)urea]	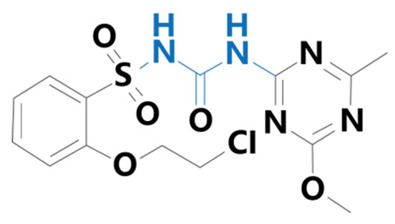	Yuanye Biotechnology Co. (Shanghai, China)	82097-50-5
Cinosulfuron	[3-(4,6-dimethoxy-1,3,5-triazin-2-yl)-1-[2-(2-methoxyethoxy)phenyl]sulfonylurea]	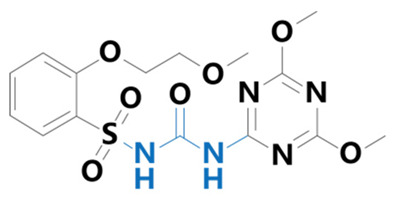	BioLabs Technology Ltd. (Beijing, China)	94593-91-6
Chlorimuron-ethyl	[2-(4-chloro-6-methoxypyrimidin-2-ylcarbamoylsulfamoyl)benzoic acid]	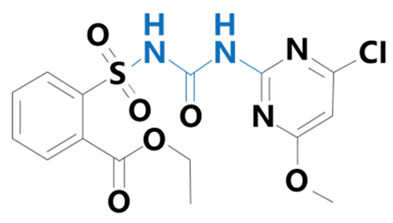	BioLabs Technology Ltd. (Beijing, China)	99283-00-8
Sulfometuron	[2-(3-(4,6-dimethylpyrimidin-2-yl)ureasulfonyl)benzoic acid]	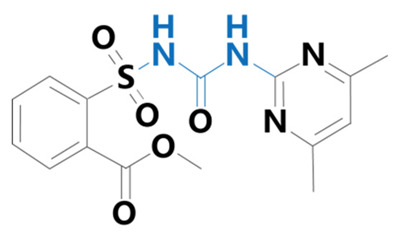	Eimage Technology Co. (Wuhan, China)	74222-97-2
Pyrazosulfuron	[5-(4,6-dimethoxypyrimidin-2-ylcarbamoylsulfamoyl)-1-methylpyrazole-4-carboxylic acid]	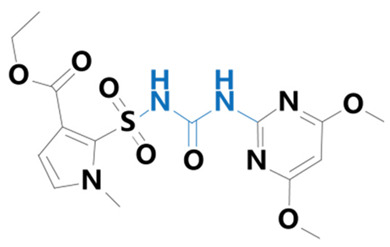	Guyan Industry Co. (Shanghai, China)	98389-04-9
Rimsulfuron	[1-(4,6-dimethoxy-2-pyrimidinyl)-3-[3-(ethylsulfonyl)-2-pyridinesulfonyl]urea]	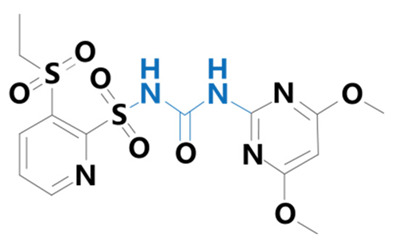	Yuanye Biotechnology Co. (Shanghai, China)	122931-48-0
Ethametsulfuron	[Methyl2-[(4-ethoxy-6-methylamino-1,3,5-triazin-2-yl)carbamoyl sulfamoyl]benzoate]	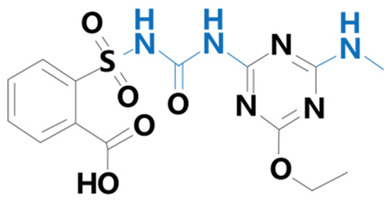	Yuanye Biotechnology Co. (Shanghai, China)	111353-84-5

## Data Availability

Data will be made available on request.

## Supplementary Materials

The following supporting information can be downloaded at: https://www.mdpi.com/article/10.3390/plants13091171/s1, Figure S1: Induced expression of apple glycosyltransferase MdUGT73CG22 protein GST tag: 26.0 KDa; MdUGT73CG22-GST: 81.0 KDa; Table S1: mRNA expression of nine glycosyltransferase genes induced by class 12 acetolactate synthase (ALS) inhibitor herbicides; Table S2: Gene primers; Table S3: Nucleotide and amino acid sequences of MdUGT73CG22.
