# Supplementary material for: First Clarification of the Involvement of Glycosyltransferase MdUGT73CG22 in the Detoxification Metabolism of Nicosulfuron in Apple
Source: Plants (Basel). 2024 Apr 23;13(9):1171. doi: 10.3390/plants13091171 (PMC11085047; doi:10.3390/plants13091171)

Figure S1. Vector construction map of the final construction. (a) Construction of prokaryotic expression vector pEGX-2T; (b) Construction of Plant Expression Vector PBI121.

a

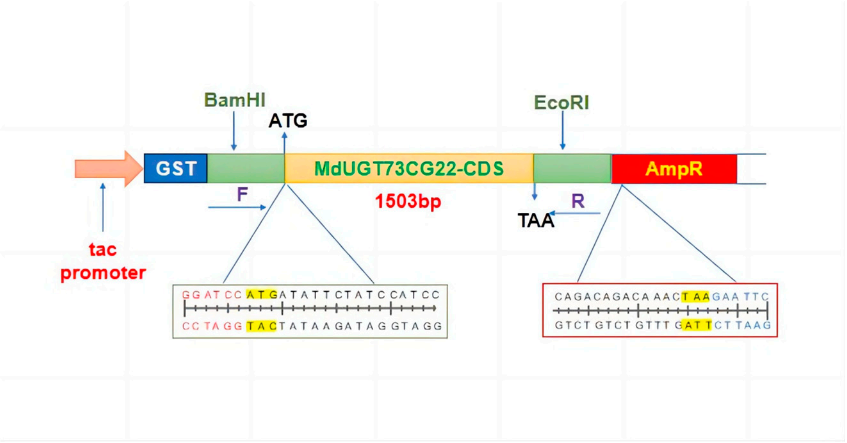

b

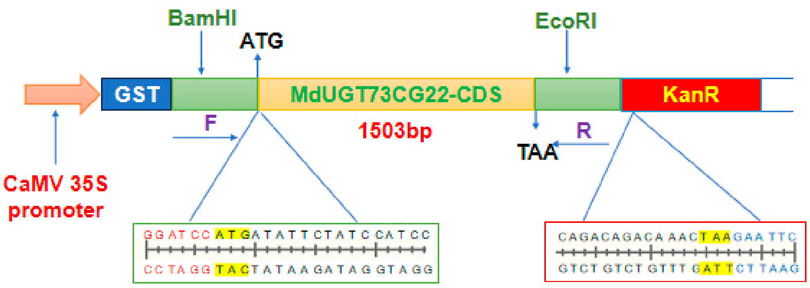

Supplement: Supplementary file 1 [file plants-13-01171-s001.zip › Supplementary Figure.pdf]
