# Supplementary material for: First Clarification of the Involvement of Glycosyltransferase MdUGT73CG22 in the Detoxification Metabolism of Nicosulfuron in Apple
Source: Plants (Basel). 2024 Apr 23;13(9):1171. doi: 10.3390/plants13091171 (PMC11085047; doi:10.3390/plants13091171)
Supplement: Supplementary file 1 [file plants-13-01171-s001.zip › Supplementary tables.pdf]

Table S1 mRNA expression of 9 glycosyltransferase genes induced by class 12 acetolactate synthase (ALS) inhibitor herbicides

Chemical compounds	Time (h)	MdUGT 73B36	MdUGT 73B37	MdUGT 73B38	MdUGT 73B39	MdUGT 73B40	MdUGT 73CG21	MdUGT 73CG22	MdUGT 73CP3	MdUGT 73AC7
Bensulfuron methyl	0	1.00 ± 0.102	1.00 ± 0.107	1.00 ± 0.114	1.00 ± 0.105	1.00 ± 0.114	1.00 ± 0.109	1.00 ± 0.110	1.00 ± 0.102	1.00 ± 0.097
	3	2.487 ± 0.249*	2.573 ± 0.284*	2.629 ± 0.218*	2.545 ± 0.197*	2.397 ± 0.183*	4.856 ± 0.378**	6.901 ± 0.425**	3.317 ± 0.243*	1.593 ± 0.227
	6	3.592 ± 0.317*	3.104 ± 0.295*	3.263 ± 0.226*	4.183 ± 0.284*	4.682 ± 0.275*	6.294 ± 0.391**	7.461 ± 0.438**	4.207 ± 0.269*	3.214 ± 0.257*
	12	2.861 ± 0.232*	3.585 ± 0.273*	2.758 ± 0.229*	3.759 ± 0.315*	3.206 ± 0.227*	5.191 ± 0.326*	5.698 ± 0.394**	3.583 ± 0.224*	2.475 ± 0.212*
	24	1.576 ± 0.085	2.408 ± 0.021*	2.174 ± 0.236*	2.103 ± 0.128	3.083 ± 0.194*	3.958 ± 0.327*	4.206 ± 0.332*	3.106 ± 0.218*	2.196 ± 0.184*
Nicosulfuron	0	1.00 ± 0.108	1.00 ± 0.112	1.00 ± 0.124	1.00 ± 0.117	1.00 ± 0.131	1.00 ± 0.121	1.00 ± 0.109	1.00 ± 0.113	1.00 ± 0.118
	3	3.594 ± 0.284*	5.302 ± 0.312*	6.394 ± 0.407**	5.390 ± 0.295*	4.495 ± 0.267*	8.683 ± 0.462**	16.289 ± 0.353**	6.283 ± 0.451**	5.253 ± 0.474**
	6	5.275 ± 0.391**	7.498 ± 0.472**	5.192 ± 0.383**	4.298 ± 0.296*	3.587 ± 0.385**	10.475 ± 0.571**	27.475 ± 0.624**	8.496 ± 0.538**	5.968 ± 0.472**
	12	5.478 ± 0.218*	6.114 ± 0.294*	3.576 ± 0.177*	3.174 ± 0.195*	2.576 ± 0.326*	7.362 ± 0.298*	22.287 ± 0.312*	7.365 ± 0.283*	6.028 ± 0.197*
	24	4.372 ± 0.329*	4.084 ± 0.276**	2.057 ± 0.215*	2.945 ± 0.195*	2.108 ± 0.226*	5.476 ± 0.321*	18.488 ± 0.274*	6.274 ± 0.325*	4.294 ± 0.269*
Thifensulfuron	0	1.00 ± 0.102	1.00 ± 0.089	1.00 ± 0.091	1.00 ± 0.113	1.00 ± 0.121	1.00 ± 0.107	1.00 ± 0.105	1.00 ± 0.124	1.00 ± 0.116
	3	2.498 ± 0.203*	3.108 ± 0.227*	3.506 ± 0.324*	2.475 ± 0.193*	4.059 ± 0.272*	5.867 ± 0.393**	9.275 ± 0.611**	7.593 ± 0.526**	5.968 ± 0.341**
	6	3.104 ± 0.202*	4.285 ± 0.271*	4.967 ± 0.314*	2.183 ± 0.156	5.290 ± 0.494**	6.193 ± 0.452**	13.573 ± 0.675**	10.437 ± 0.518**	4.677 ± 0.305*
	12	3.587 ± 0.226*	5.227 ± 0.313*	3.264 ± 0.237*	1.941 ± 0.106	3.924 ± 0.191*	7.958 ± 0.425**	10.486 ± 0.608**	7.079 ± 0.436**	3.86 ± 0.284*
	24	1.951 ± 0.104	3.296 ± 0.192*	2.813 ± 0.175	1.756 ± 0.126	2.856 ± 0.174	4.204 ± 0.309*	7.497 ± 0.516**	5.398 ± 0.347**	2.913 ± 0.206*
Chlorsulfuron	0	1.00 ± 0.109	1.00 ± 0.105	1.00 ± 0.102	1.00 ± 0.095	1.00 ± 0.097	1.00 ± 0.099	1.00 ± 0.101	1.00 ± 0.112	1.00 ± 0.106
	3	1.958 ± 0.116	2.057 ± 0.184*	3.912 ± 0.253*	3.205 ± 0.157	2.497 ± 0.195*	3.576 ± 0.264*	4.587 ± 0.295*	3.857 ± 0.291*	3.595 ± 0.246*
	6	2.475 ± 0.206*	3.186 ± 0.292*	5.364 ± 0.427**	4.592 ± 0.314*	3.185 ± 0.196*	3.958 ± 0.257*	5.920 ± 0.475**	3.184 ± 0.246*	4.952 ± 0.395**
	12	3.049 ± 0.273*	4.208 ± 0.377**	6.721 ± 0.426**	5.365 ± 0.348**	3.596 ± 0.207*	4.697 ± 0.394**	4.867 ± 0.326**	2.172 ± 0.195*	2.954 ± 0.212*
	24	2.176 ±	3.756 ±	4.598 ±	6.358 ±	3.284 ±	3.458 ±	4.103 ±	1.485 ±	2.193 ±

		0.225*	0.298*	0.342**	0.487**	0.192*	0.242*	0.375**	0.107	0.214*
Metsulfuron	0	1.00 ± 0.095	1.00 ± 0.098	1.00 ± 0.112	1.00 ± 0.114	1.00 ± 0.112	1.00 ± 0.109	1.00 ± 0.106	1.00 ± 0.103	1.00 ± 0.089
	3	2.986 ± 0.224*	3.586 ± 0.312*	4.598 ± 0.389**	3.943 ± 0.316*	4.523 ± 0.328*	3.598 ± 0.314*	5.393 ± 0.407**	3.718 ± 0.307*	2.746 ± 0.224*
	6	3.697 ± 0.313*	4.592 ± 0.339**	3.576 ± 0.284*	4.695 ± 0.397**	3.209 ± 0.247*	3.193 ± 0.225*	6.892 ± 0.416**	2.905 ± 0.217*	2.403 ± 0.211*
	12	2.465 ± 0.178*	5.258 ± 0.472**	4.052 ± 0.345**	3.291 ± 0.283*	2.985 ± 0.226*	2.857 ± 0.279*	5.304 ± 0.264**	2.164 ± 0.172*	1.725 ± 0.153
	24	1.758 ± 0.126	3.591 ± 0.197*	2.527 ± 0.173*	2.476 ± 0.192*	1.756 ± 0.086	2.475 ± 0.158*	3.068 ± 0.247*	1.957 ± 0.139	1.590 ± 0.103
Triasulfuron	0	1.00 ± 0.101	1.00 ± 0.114	1.00 ± 0.118	1.00 ± 0.112	1.00 ± 0.093	1.00 ± 0.095	1.00 ± 0.097	1.00 ± 0.102	1.00 ± 0.105
	3	3.194 ± 0.287*	2.457 ± 0.205*	1.598 ± 0.117	3.967 ± 0.286*	2.941 ± 0.254*	2.847 ± 0.212*	4.596 ± 0.339**	4.209 ± 0.327*	5.212 ± 0.356**
	6	4.875 ± 0.384**	3.269 ± 0.291*	2.587 ± 0.216*	5.182 ± 0.317*	4.952 ± 0.338**	3.492 ± 0.286*	5.291 ± 0.299*	3.586 ± 0.312*	3.298 ± 0.300*
	12	2.986 ± 0.183*	2.173 ± 0.179*	3.736 ± 0.242*	4.295 ± 0.309*	3.687 ± 0.273*	3.103 ± 0.295*	3.059 ± 0.178*	2.375 ± 0.168*	2.985 ± 0.217*
	24	2.473 ± 0.195*	2.048 ± 0.181*	2.592 ± 0.213*	3.698 ± 0.226*	3.139 ± 0.295*	2.171 ± 0.204*	2.506 ± 0.194*	2.109 ± 0.183*	2.174 ± 0.169*
Cinosulfuron	0	1.00 ± 0.126	1.00 ± 0.118	1.00 ± 0.115	1.00 ± 0.113	1.00 ± 0.109	1.00 ± 0.110	1.00 ± 0.101	1.00 ± 0.098	1.00 ± 0.104
	3	4.295 ± 0.371**	3.941 ± 0.312*	5.282 ± 0.420**	4.287 ± 0.323*	3.953 ± 0.297*	3.917 ± 0.248*	4.576 ± 0.374**	3.961 ± 0.392*	3.738 ± 0.329*
	6	5.957 ± 0.414**	3.276 ± 0.293*	3.953 ± 0.317*	3.952 ± 0.287*	3.274 ± 0.306*	3.094 ± 0.232*	5.689 ± 0.513**	3.295 ± 0.319*	2.584 ± 0.209**
	12	3.541 ± 0.272*	2.478 ± 0.220*	3.747 ± 0.295*	3.648 ± 0.262*	2.792 ± 0.245*	2.479 ± 0.216*	4.275 ± 0.335**	2.472 ± 0.224*	2.103 ± 0.182*
	24	3.208 ± 0.271*	2.103 ± 0.192*	3.029 ± 0.325*	3.209 ± 0.247*	2.165 ± 0.197*	2.153 ± 0.204*	3.136 ± 0.295*	2.109 ± 0.157*	1.712 ± 0.143
Chlorimuron-ethyl	0	1.00 ± 0.112	1.00 ± 0.115	1.00 ± 0.108	1.00 ± 0.114	1.00 ± 0.109	1.00 ± 0.110	1.00 ± 0.113	1.00 ± 0.121	1.00 ± 0.117
	3	4.495 ± 0.325*	5.067 ± 0.419**	3.587 ± 0.272*	4.693 ± 0.401**	4.142 ± 0.275*	3.673 ± 0.322*	4.593 ± 0.373**	3.103 ± 0.227*	2.757 ± 0.206*
	6	3.957 ± 0.328*	3.953 ± 0.291*	2.746 ± 0.224*	3.405 ± 0.325*	2.498 ± 0.229*	2.487 ± 0.195*	3.193 ± 0.225*	2.164 ± 0.127*	2.536 ± 0.159*
	12	3.162 ± 0.217*	3.714 ± 0.302*	2.293 ± 0.206*	2.381 ± 0.236*	2.103 ± 0.179*	2.212 ± 0.184*	2.597 ± 0.210*	1.475 ± 0.126	2.183 ± 0.194*
	24	2.373 ± 0.225*	2.673 ± 0.245*	1.305 ± 0.184	2.049 ± 0.197*	1.325 ± 0.102	1.475 ± 0.136	2.104 ± 0.198*	1.028 ± 0.113	1.472 ± 0.157
Sulfometuron	0	1.00 ± 0.108	1.00 ± 0.114	1.00 ± 0.112	1.00 ± 0.109	1.00 ± 0.110	1.00 ± 0.106	1.00 ± 0.111	1.00 ± 0.108	1.00 ± 0.105
	3	2.489 ±	2.967 ±	3.638 ±	2.574 ±	2.579 ±	3.952 ±	4.476 ±	3.113 ±	2.465 ±

		0.184*	0.219*	0.247*	0.195*	0.206*	0.324*	0.308*	0.235*	0.213*
	6	1.487 ± 0.105	2.572 ± 0.225*	2.954 ± 0.236*	2.135 ± 0.189*	2.163 ± 0.195*	3.174 ± 0.228*	3.102 ± 0.274*	2.712 ± 0.225*	2.104 ± 0.125*
	12	1.264 ± 0.117	2.469 ± 0.192*	2.746 ± 0.227*	1.846 ± 0.158	1.765 ± 0.154	2.533 ± 0.252*	2.528 ± 0.212*	2.384 ± 0.225*	1.675 ± 0.119
	24	1.141 ± 0.103	2.103 ± 0.192*	2.429 ± 0.213*	1.457 ± 0.136	1.354 ± 0.121	2.106 ± 0.175*	2.294 ± 0.193*	1.948 ± 0.166	1.382 ± 0.121
	0	1.00 ± 0.112	1.00 ± 0.115	1.00 ± 0.109	1.00 ± 0.106	1.00 ± 0.107	1.00 ± 0.110	1.00 ± 0.094	1.00 ± 0.096	1.00 ± 0.098
	3	3.193 ± 0.271*	4.285 ± 0.336**	3.853 ± 0.292*	2.048 ± 0.164*	2.375 ± 0.201*	2.598 ± 0.324*	3.751 ± 0.327*	3.105 ± 0.253*	2.968 ± 0.283*
Pyrazosulfuron	6	4.284 ± 0.306*	3.162 ± 0.291*	3.476 ± 0.269*	1.574 ± 0.126	2.103 ± 0.184*	2.346 ± 0.205*	4.548 ± 0.320*	1.752 ± 0.154	2.457 ± 0.213*
	12	3.481 ± 0.292*	2.476 ± 0.263*	3.102 ± 0.274*	1.395 ± 0.113	1.358 ± 0.125	2.104 ± 0.176*	2.958 ± 0.225*	1.534 ± 0.137	2.182 ± 0.175*
	24	2.958 ± 0.253*	2.103 ± 0.185*	2.174 ± 0.192*	1.202 ± 0.103	1.249 ± 0.115	1.467 ± 0.138	2.472 ± 0.213*	1.372 ± 0.115	1.563 ± 0.147
	0	1.00 ± 0.105	1.00 ± 0.102	1.00 ± 0.112	1.00 ± 0.114	1.00 ± 0.117	1.00 ± 0.121	1.00 ± 0.125	1.00 ± 0.116	1.00 ± 0.118
	3	3.597 ± 0.285*	2.453 ± 0.221*	2.587 ± 0.209*	3.116 ± 0.237*	2.498 ± 0.223*	3.587 ± 0.336*	3.759 ± 0.294*	3.285 ± 0.246*	2.574 ± 0.209*
Rimsulfuron	6	4.483 ± 0.317*	3.198 ± 0.296*	2.140 ± 0.182*	2.585 ± 0.199*	2.102 ± 0.164*	2.954 ± 0.182*	3.106 ± 0.274*	2.954 ± 0.278*	2.148 ± 0.195*
	12	5.648 ± 0.427**	2.284 ± 0.228*	1.483 ± 0.135	2.147 ± 0.186*	1.365 ± 0.126	2.463 ± 0.217*	2.587 ± 0.226*	2.756 ± 0.249*	1.476 ± 0.139
	24	3.174 ± 0.225*	1.035 ± 0.108	1.205 ± 0.157	1.934 ± 0.165	1.241 ± 0.113	2.212 ± 0.148*	2.142 ± 0.183*	2.392 ± 0.193*	1.291 ± 0.115
	0	1.00 ± 0.109	1.00 ± 0.112	1.00 ± 0.109	1.00 ± 0.114	1.00 ± 0.112	1.00 ± 0.106	1.00 ± 0.095	1.00 ± 0.098	1.00 ± 0.097
	3	2.498 ± 0.205*	2.962 ± 0.217*	3.593 ± 0.236*	4.235 ± 0.293*	3.594 ± 0.225*	4.206 ± 0.326*	5.205 ± 0.417**	4.698 ± 0.308*	3.574 ± 0.371*
Ethametsulfuron	6	3.194 ± 0.283*	4.681 ± 0.305*	5.673 ± 0.401**	3.597 ± 0.312*	4.182 ± 0.381**	3.384 ± 0.347**	4.746 ± 0.358**	3.567 ± 0.251*	2.473 ± 0.228*
	12	2.971 ± 0.283*	3.587 ± 0.245*	3.275 ± 0.221*	3.104 ± 0.273*	2.053 ± 0.152*	2.915 ± 0.238*	3.179 ± 0.225*	3.193 ± 0.253*	2.145 ± 0.182*
	24	2.752 ± 0.226*	2.583 ± 0.218*	2.597 ± 0.232*	2.953 ± 0.194*	1.427 ± 0.101	1.759 ± 0.112	2.487 ± 0.124	2.576 ± 0.136	1.847 ± 0.129

Table S2 Gene Primers

Gene name	Primer sequence	Function
MdUGT73B36	F: GTCGAAATCAAAACCATC<br>R: ACTTTGCAGCTGCATCAG	For Real-time PCR
MdUGT73B37	F: GGAGAAAGAGGATTGGCTG<br>R: CTCGTTGTAAAACTGCTCC	For Real-time PCR
MdUGT73B38	F: TAGGATGGAAGGGAAAGG<br>R: CCATTTTTGAGCACCAAC	For Real-time PCR
MdUGT73B39	F: GACACGAGCCCAAGTTCC<br>R: GCTTTCATCCCAAGCACG	For Real-time PCR
MdUGT73B40	F: GCTGAGAAAACGAGCTATGG<br>R: TGAATTTAGCCATGCTTCC	For Real-time PCR
MdUGT73CG21	F: GGAGGGTTCTTAACACACTG<br>R: CACCAACACCCCAATCTTC	For Real-time PCR
MdUGT73CG22	F: CCATCGTCACTACGCAGCAC<br>R: CTTCTCTGCTTGTTGTTGC	For Real-time PCR
MdUGT73CP3	F: TCTAGACCTCCTGCTTCATATATGAT<br>R: GGATCCCTCCTCTCCTTAGTTTGTCTG	For Real-time PCR
MdUGT73CR7	F: GGATTGCAGATGTCACTAAC<br>R: CACTCTAGAGCCTCATTGAG	For Real-time PCR
MdUGT73CG22	F: GGATCCATGATATTCTATCCATCC<br>R: GAATTCTTAGTTTGTCTGTCTG	Full-length gene amplification

Table S3 Nucleotide and amino acid sequences of MdUGT73CG22

cDNA sequences

AGGAAGCAAGTTATGTGAAGTGGCTAAACAGAGTCCACATAACTGAGTAGAGTGAAAT
GTTAGGATAAGAACACCAATAAATTTGTAAGCTCTCACGTCAGCAGCGGCAAATTGCAT
CATTGTCCCGTAGCAAGAGAGAGATGCTATATCCCTCGAGTAACAAGCGAACCTCCTG
CTTCATAT**ATG**ATATTCTATCCATCCAATTTTCTTCCCAATACCATGGCGTCCGGAGAGCC

CCAGCTTCACTTTGTTATGTTTCCTTTCATGGCCCAAGGCCACATGATACCCATGATCGA<br>CATCGCTAGACTCTTGGCGCAAAGAGGCATCATCATCACCATCGTCACTACGCAGCAC<br>AATGCGGCGCGATTTCAAAACGTTTTGACTCGTGCTGTGCAGTCGGGCCTCCAAATGA<br>GGGTAGCCCTACTAGAATTTCCATGCGAAAAAGCGGGGTTGCCTGCTGGGTGTGAGAA<br>CCTTGACCTGCTTCCGTCGCATGGATTGGCGGCGCACTTCTTTTTTGCAACGGGTCTGT<br>TGCAACAACAAGCAGAGAAGTTGTTTGAAGAGTTAACCCCCGAGCCAAGCTGCATATT<br>CTCCGATATGTGTTTTCCGTGGACGGTGAAAATTTCCCACAAGTTTAACATTCCAAGGA<br>TATCTTTCAGTGGATTCTGCACATTTTGTCTCCTTTGCATTAACAAACTTCAGGCGTCGA<br>AAGTTCATGAGAATTTAAGCTCTGAAACGGAGTACTTTGTTGTGCCTGACTTGCCTGAT<br>CGAATTGAGGCCACGAAAGCTCAGCTGCCAGGCCCCCTGACTCCAAGTATGTCAGGGT<br>TTTTGGATAAAATTGTTGCAGCTGAATTGGAAACATTTGGGATAATCATGAATACTTTTG<br>AAGAGTTGGAGCCGGCATATATTGAAGCGTACAAGAAGACGGCGAAAGTCTGGTGCAT<br>TGGCCCAGCTTCACTGAGCAACAAAGATGACTTGGACAAGGCGCAGAGGGGTAACAA<br>GGCCTCCATTGATGAACAGCACTGCTTGAAGTGGCTTGATTCTTGGGAACCAAGTTCT<br>GTAATTTATGCTTGCCTCGGAAGTTTATGCAATCTTGTGTGTGAACAATTGATAGAGCTT<br>GGACTGGCCTTAGAGGCATCGAACAAAACGTTCATTTGGGTTGTAAGGGGATGCAGTC<br>AATCAGAAGAGTTGGAAAAATGGATTTCTGAGAGTGAATTTGAAGAAAGAACGAAGG<br>GAAGGAGCCTCGTTATTTGGGGATGGGCTCCGCAAACACTCATATTGTCACACCGTGC<br>AGTTGGGGGGTTCTTAACACATTGTGGATGGAACTCGATAATGGAAGGGATATGTGCCG<br>GGCTGCCCTTGATCACATGGCCACTGTTTGGGGACCAGTTCCTTAATGAAAAACTAGTT<br>GTGCAGGTTCTTAAAATTGCGGTGAGCGTTGGGGTGGAGTATCCGGTAAAGTGGGGAG<br>AGGAAGAGAAGATTGGCGTGCTGGTGAAAAGGGAAAATGTAACAAAAGCGATAGAG<br>AAGGTGATGGATGGAGAAGAAAGTGAAGGGAGAAGAGAGAGAGCCAGAGAGTTTGG<br>AGAGATGGCAAAGAGAGCAGCAGAAGAAGGAGGATCGTCTCACGTCAACGTTTCACA<br>GCTTATCCAAGATATCGTGCAACAAAGTAGTAATGGCAGACAGACAAAC**TAA**GGAGAG<br>GAGAATACAAGGGACTTGCTGCTCCATCTCAACTGAAAGAGCTTCTTGTCTCATGTACT<br>GTAAACATGTCATTTGTTGCAAAATTAGTTTTCTTTTCTTTTTCTTTCTCTGTAGATTTAT<br>ACTTCAGAAACAATTGATACACCTGCTTTACTTA
Amino acid sequences

MIFYPSNFLPNTMASGEPQLHFVMFPFMAQGHMIPMIDIARLLAQRGIIITIVTTQHNAARF
QNVLTRAVQSGLQMRVALLEFPCEKAGLPAGCENLDLLPSHGLAAHFFFATGLLQQQAEK
LFEELTPEPSCIFSDMCFPWTVKISHKFNIPRISFSGFCTFCLLCINKLQASKVHENLSSETEY
FVVPDLPDRIEATKAQLPGPLTPSMSGFLDKIVAAELETFGIIMNTFEELEPAYIEAYKKTAK
VWCIGPASLSNKDDLDKAQRGNKASIDEQHCLKWLDSWEPSSVIYACLGSLCNLVCEQLI
ELGLALEASNKTFIWVVRGCSQSEELEKWISESEFEERTKGRSLVIWGWAPQTLILSHRAV
GGFLTHCGWNSIMEGICAGLPLITWPLFGDQFLNEKLVVQVLKIAVSVGVEYPVKWGEEE
KIGVLVKRENVTKAIEKVMDGEESEGRRERAREFGEMAKRAAEEGGSSHVNVSQLIQDIV
QQSSNGRQTN
